# Advantages and Challenges of Using Telehealth for Home-Based Palliative Care: Systematic Mixed Studies Review

**DOI:** 10.2196/43684

**Published:** 2023-03-13

**Authors:** Simen A Steindal, Andréa Aparecida Gonçalves Nes, Tove E Godskesen, Heidi Holmen, Anette Winger, Jane Österlind, Alfhild Dihle, Anna Klarare

**Affiliations:** 1 Lovisenberg Diaconal University College Oslo Norway; 2 Institute of Nursing Faculty of Health Studies VID Specialized University Oslo Norway; 3 Department of Health Care Sciences, Palliative Research Centre Marie Cederschiöld University Stockholm Sweden; 4 Centre for Research Ethics & Bioethics Uppsala University Uppsala Sweden; 5 Department of Nursing and Health Promotion Faculty of Health Sciences Oslo Metropolitan University Oslo Norway; 6 The Intervention Centre Oslo University Hospital Oslo Norway; 7 Healthcare Services and e-Health Department for Women’s and Children’s Health Uppsala University Uppsala Sweden

**Keywords:** digital health, eHealth, health care technology, home-based palliative care, review, systematic mixed studies review, telemedicine, mobile phone

## Abstract

**Background:**

Owing to the increasing number of people with palliative care needs and the current shortage of health care professionals (HCPs), providing quality palliative care has become challenging. Telehealth could enable patients to spend as much time as possible at home. However, no previous systematic mixed studies reviews have synthesized evidence on patients’ experiences of the advantages and challenges of telehealth in home-based palliative care.

**Objective:**

In this systematic mixed studies review, we aimed to critically appraise and synthesize the findings from studies that investigated patients’ use of telehealth in home-based palliative care, focusing on the advantages and challenges experienced by patients.

**Methods:**

This is a systematic mixed studies review with a convergent design. The review is reported according to the PRISMA (Preferred Reporting Items for Systematic Reviews and Meta-Analyses) statement. A systematic search was performed in the following databases: Allied and Complementary Medicine Database, CINAHL, Cochrane Central Register of Controlled Trials, Embase, Latin American and Caribbean Health Sciences Literature, MEDLINE, PsycInfo, and Web of Science. The inclusion criteria were as follows: studies using quantitative, qualitative, or mixed methods; studies that investigated the experience of using telehealth with follow-up from HCPs of home-based patients aged ≥18; studies published between January 2010 and June 2022; and studies published in Norwegian, Danish, Swedish, English, Portuguese, or Spanish in peer-reviewed journals. Five pairs of authors independently assessed eligibility of the studies, appraised methodological quality, and extracted data. The data were synthesized using thematic synthesis.

**Results:**

This systematic mixed studies review included 41 reports from 40 studies. The following 4 analytical themes were synthesized: potential for a support system and self-governance at home; visibility supports interpersonal relationships and a joint understanding of care needs; optimized information flow facilitates tailoring of remote caring practices; and technology, relationships, and complexity as perpetual obstacles in telehealth.

**Conclusions:**

The advantages of telehealth were that patients experience a potential support system that could enable them to remain at home, and the visual features of telehealth enable them to build interpersonal relationships with HCPs over time. Self-reporting provides HCPs with information about symptoms and circumstances that facilitates tailoring care to specific patients. Challenges with the use of telehealth were related to barriers to technology use and inflexible reporting of complex and fluctuating symptoms and circumstances using electronic questionnaires. Few studies have included the self-reporting of existential or spiritual concerns, emotions, and well-being. Some patients perceived telehealth as intrusive and a threat to their privacy at home. To optimize the advantages and minimize the challenges with the use of telehealth in home-based palliative care, future research should include users in the design and development process.

## Introduction

### Background

Palliative care (PC) can be introduced early in the illness trajectory of patients with life-threatening illnesses, and it aims to alleviate burdensome symptoms and optimize patient quality of life [1-3]. Recent literature has emphasized the importance of a patient-centered approach in PC, in which health care professionals (HCPs) are responsive to individual patient needs and preferences and ensure that the provision of care is guided by the patient’s preferences [4,5]. The 6S-model is one such approach for person-centered PC. Self-image (identity) is the core concept that underlines the importance of having a good view of the self [4]. To preserve self-image, optimal symptom management is essential. Social relationships reflect the person’s social needs such as connections with others, whereas self-determination (autonomy) reflects the need to live according to their own values and beliefs [4]. Patients with PC needs often want to retain their self-determination, live a meaningful life, and live as normally as possible, and many of these patients want to spend as much time as possible at home [6,7]. Feeling secure seems to be a prerequisite for patients to be able to spend time at home [8,9]. For instance, keeping promises, being reliable, and creating partnerships are actions by HCPs in specialist palliative home care teams that patients experience as important in meeting their needs [10]. Patients with PC needs have emphasized that an alert and assertive attitude on their own part is important to ensure that they receive the care they need from municipal HCPs. However, these patients are concerned about other patients who are less vigilant regarding the care they receive or who have no family to support them [11].

There are concerns regarding both the expected increase in patients with PC needs [12] and the future workforce strain in PC contexts, which might result in insufficient numbers of HCPs to cover the shortfall [13]. The United Nations underscores patients’ rights to access quality health care services [14]. The use of telehealth in home-based PC may be one model to address the above-mentioned challenges. Telehealth can enable HCPs to communicate and follow-up with home-based patients remotely and reduce HCPs’ need to travel. This could enable them to use time and resources more effectively [15].

Telehealth is defined as “the provision of health care remotely by means of a variety of telecommunication tools” [16]. Technology, including visual and auditory components such as video-based technology for teleconsultations and written components such as apps on smartphones and tablets for remote monitoring of patients’ symptoms, are frequently used in home-based PC [17]. Telehealth can be delivered in an asynchronous (passive) mode, which is a form of interaction or communication that does not need an instant response from the recipient or in a synchronous (interactive) mode which is a form of interaction that involves exchange of messages or information between patients and HCPs in real time [18].

The use of telehealth may empower patients to manage their illness, improve their quality of life, improve their access to home-based PC services, reduce unnecessary hospitalization, and reduce hospital care costs [19,20]. The use of telehealth may change how PC is delivered [21]. In the context of PC, the relationship between patients and HCPs is of great importance. However, there are concerns about how telehealth may affect such relationships and patients’ ability to report existential and psychosocial needs using telehealth [21]. Furthermore, lack of experience and adequate knowledge about PC among home care HCPs such as nurses, physiotherapists, and occupational therapists may be a challenge when using telehealth in home-based PC [22].

Systematic meta-reviews have investigated the role and effect of telehealth interventions in PC [23] and identified telehealth technologies that have been evaluated for supporting the timely assessment and management of people with PC needs at home [24]. Previous systematic reviews have investigated the use of telehealth for children receiving PC [25,26]. Another systematic review investigated the existing information and communication technology systems intended to support pain management in patients with cancer who received PC. However, the included studies were not limited to patients’ experiences of using these information and communication technology systems at home [27]. Other systematic reviews have investigated patient-reported outcomes, such as symptoms, quality of life, and satisfaction [20], as well as experiences with video communication [28] and the effectiveness of telehealth interventions in relation to the information needs of people involved in the PC process [29]. Patients’ experiences of using telehealth in PC have been investigated in a scoping review [17] and an integrative review [30], and the findings suggest that telehealth promoted and enhanced communication with HCPs, empowered participation and care governance [30], and enhanced patients’ feelings of safety and security [17]. However, because of the review designs, inferences and recommendations for policy and practice cannot be conclusively stated based on their findings. Furthermore, future systematic reviews should address the so far neglected negative aspects of telehealth in home-based PC [17].

### Objective

Our initial literature searches revealed that no systematic mixed studies reviews have synthesized evidence on patients’ experiences of the advantages and challenges of using telehealth in home-based PC. Such reviews could enable a comprehensive and rich understanding of a complex intervention [31] such as telehealth in home-based PC and could identify barriers to and facilitators for the adoption of technology, as these requirements could influence the design, use, and function of the developed technology [32]. Consequently, this systematic mixed studies review aimed to critically appraise and synthesize the findings from studies that investigated patients’ use of telehealth in home-based PC. We were guided by the following research question: What do patients experience as the advantages and challenges of using telehealth in home-based PC?

## Methods

### Design

This systematic mixed studies review used a convergent design [31] and included studies regardless of the method and study design*.* In a convergent design, the results of the included studies are integrated using qualitative data transformation techniques, and quantitative data are transformed into qualitative findings [31]. The review was reported according to the PRISMA (Preferred Reporting Items for Systematic Reviews and Meta-Analyses) statement [33] (Multimedia Appendix 1). Deviations from the published protocol [34] are described in Multimedia Appendix 2.

### Eligibility Criteria

The eligibility criteria are listed in Table 1. The included studies were limited to the period from the beginning of 2010. The period was chosen to include studies that used modern and relevant telehealth services. Telephonic follow-up was excluded as we wanted to investigate patients’ experiences of using modern telehealth. The languages were limited to those that the authors spoke and understood.

**Table 1:** Eligibility criteria.

Criteria	Inclusion	Exclusion
Types of studies	Any type of quantitative, qualitative, or mixed methods studies on the phenomenon of interest published in peer-reviewed journals	Any type of review, PhD theses, conference abstracts, editorials, comments, and letters
Period	From January 1, 2010, to June 3, 2022	Before January 1, 2010, and after June 3, 2022
Languages	Norwegian, Danish, Swedish, English, Portuguese, and Spanish	All other languages
Participants	Home-based patients aged 18 years or older in a PC^a^ trajectory, regardless of diagnosis	Patients 17 years or younger, not in a PC trajectory, or using telehealth in a hospital setting, hospice, or nursing home
Phenomenon of interest	Home-based patients’ experience of using telehealth with follow-up from HCPs^b^	Home-based patients’ experience of using telehealth without follow-up from HCPs; or, patients’ experience of using telehealth in a hospital, hospice, or nursing home. Only telephone follow-up from HCPs at home
Outcomes	Patients’ subjective and objective outcomes	Proxy-reported outcomes

^a^PC: palliative care.

^b^HCP: health care professional.

### Search Strategy

A systematic search was performed using the following databases: the Allied and Complementary Medicine Database, CINAHL, the Cochrane Central Register of Controlled Trials, Embase, Latin American and Caribbean Health Sciences Literature, MEDLINE, PsycInfo, and the Web of Science. The search was performed on June 25, 2020, and was updated on June 3, 2022. The search strategy was built in MEDLINE by an experienced research librarian (FS), AW and SAS using text words and subject headings adopted for each of the databases. The search strategy consisted of 2 elements, namely PC and telehealth (Multimedia Appendix 3). A second research librarian (SKC) critically reviewed the search strategy according to the peer review of the electronic search strategies checklist [35]. We also conducted backward and forward citation searches as well as a manual search of JMIR journals.

### Study Selection

The research librarian (FS) imported the identified records to EndNote (Clarivate) for the removal of duplicate studies. The records were migrated to Rayyan (Rayyan Systems Inc) for the storage and facilitation of blinding during the screening process of titles and abstracts [36]. In total, 5 pairs of authors (AK and SAS, AAGN and SAS, AW and HH, TEG and Susanne Lind, and AD and JÖ) independently assessed whether the titles, abstracts, and full-text reports met the inclusion criteria. An additional author conducted an independent assessment when there was a doubt about whether a report should be included, and an agreement was reached based on discussion and negotiated consensus [37].

### Appraisal of Methodological Quality

On the basis of the study design, the methodological quality of the included studies was independently appraised by the same pairs of authors using the Mixed Methods Appraisal Tool (version 2018) [38]. This tool can be used in systematic mixed studies reviews to appraise the methodological quality of 5 different study designs, that is, qualitative, quantitative randomized controlled trials, quantitative nonrandomized, quantitative descriptive, and mixed method studies. The Mixed Methods Appraisal Tool contains 2 initial screening questions, and then each category (based on the study design) contains 5 criteria that are rated using “yes,” “no,” or “can’t tell” [38].

### Data Extraction

Data were extracted from the included reports using a standardized data collection form that included the following data: year of publication, country of origin, the aim of the study, study population and sample size, theoretical framework for the telehealth intervention, telehealth application and delivery mode, design and methods, and findings related to the research questions of the review. The included articles were equally divided among the pairs of authors: 1 author extracted the data, while the other checked the data accuracy.

### Data Synthesis

The data were synthesized using inductive thematic synthesis [31,39], which has previously been used in systematic mixed studies reviews with a convergent design [25,40]. NVivo (version 12; QSR International) was used for storage and analysis in the first step of data synthesis. Quantitative data were transformed into a qualitative format [31]: numerical data presented in tables and figures were described in words and supported by the authors’ description of the results from the result sections of the included reports.

In the first step, the result sections of the included reports were read several times to obtain an understanding of the material as a whole. Then, the result section of each report was coded line by line using NVivo, according to its content and meaning. All the codes, including the corresponding meaning units, were transported to Microsoft Word (Microsoft Inc) documents, where they were further developed and refined by collapsing and splitting the codes. In the second step, guided by the research question, the codes were sorted into descriptive themes close to the findings of the included reports based on similarities and differences between the codes. In the third step, to generate analytical themes, the descriptive themes were interpreted and abstracted, guided by the research question [39,41].

## Results

### Overview

**Figure 1:**
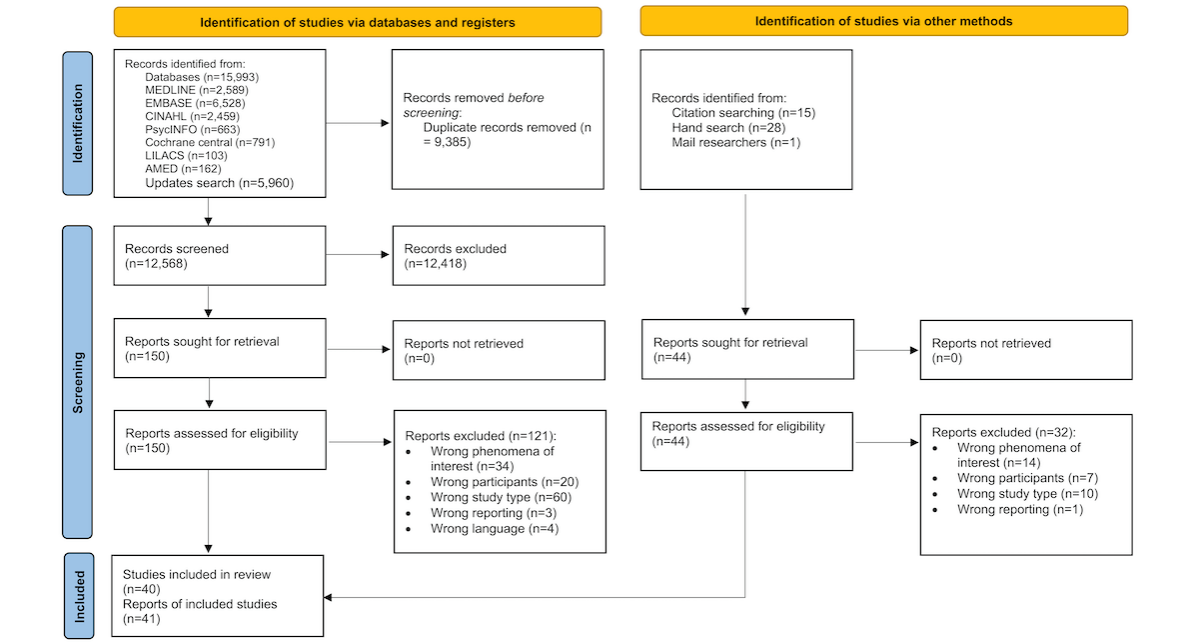
Summary of the selection of studies. AMED: Allied and Complementary Medicine Database; CENTRAL: Cochrane Central Register of Controlled Trials; LILACS: Latin American and Caribbean Health Sciences Literature.

The database searches identified 21,953 studies. Of these, after the removal of duplicates (9385/21,953, 42.75%), we screened the titles and abstracts of 57.25% (12,568/21,953) of studies. On the basis of the eligibility criteria, we read 1.19% (150/12,568) of full-text reports, of which 80.7% (121/150) of reports were excluded and 29 (19.3%) reports from 28 studies were included. In addition, 12 reports were identified through backward and forward citation searches. The reasons for the exclusion of full-text reports are shown in Figure 1.

### Description of the Included Studies

A total of 41 reports from 40 studies were included. Two reports were from the same qualitative study [42,43]. The included studies were conducted in Australia (n=6), Austria (n=1), Brazil (n=1), Canada (n=4), Chile (n=1), Denmark (n=1), Finland (n=1), the Netherlands (n=5), Portugal (n=1), Sweden (n=2), Tanzania (n=1), the United Kingdom (n=7), and the United States (n=9). The sample sizes of the included studies ranged from 1 to 234 participants. Studies included patients with cancer (n=22, 55%) and different life-limiting illnesses, including both cancer and noncancer diagnoses (n=8, 20%), motor neuron disease (n=4, 10%), heart failure (n=2, 5%), and chronic obstructive pulmonary disease (n=1, 2%). Diagnoses were not described in 3 (8%) of the studies. Overall, 25% (n=10) of studies were qualitative studies [42-52]; 8% (n=3) were quantitative randomized trials [53-55]; 30% (n=12) of studies were quantitative nonrandomized trials [56-67]; 10% (n=4) of studies were quantitative descriptive studies [68-71]; and 28% (n=11) were mixed methods or multimethod studies [72-82]. Furthermore, 18% (n=7) of studies described the theoretical framework for telehealth applications [49,50,57,59,62,74,76].

The most frequent modality for the delivery of telehealth was video using the synchronous mode, which was mostly used for teleconsultations (n=19), and remote monitoring using the asynchronous mode for monitoring and management of symptoms (n=13). In 5 studies, video using synchronous mode and remote monitoring using asynchronous mode were used, whereas in 1 study, both video and remote monitoring using synchronous mode were used, for example, to assess patients’ symptoms during the video consultation. In 2 studies, telehealth was delivered using web portals that included remote written communication using the asynchronous mode, for example, for dignity therapy, while 1 study used a webinar platform that included audio and written synchronous modes. The characteristics of the included studies are shown in Multimedia Appendix 4.

### Appraisal of the Methodological Quality of the Included Studies

Most of the included qualitative studies (9/10, 90%) fulfilled all the quality criteria. Overall, 19 studies used a quantitative method; however, only 16% (n=3) of these studies used a randomized controlled trial design. Several of the mixed methods or multimethods studies did not sufficiently integrate or describe the integration of the quantitative and the qualitative components (Multimedia Appendix 5 [44-82]).

### Thematic Synthesis

To answer our research question, “What do patients experience as the advantages and challenges of using telehealth in home-based PC,” we synthesized the following 4 analytical themes: potential for a support system and self-governance at home; visibility supports interpersonal relationships and a joint understanding of care needs; optimized information flow facilitates tailoring of remote caring practices; and technology, relationships, and complexity as perpetual obstacles in telehealth. The descriptive and analytical themes are described in Table 2.

**Table 2:** Description of descriptive themes and analytical themes.

Descriptive themes	Analytical themes
Technology endorsed access to health care professional support at home Feasible, acceptable, and useful technology	Potential for a support system and self-governance at home
Building relationships and connections over time using video conferences Understanding symptoms and circumstances remotely using video conferences	Visibility supports interpersonal relationships and a joint understanding of care needs
Self-reporting enables management of symptoms and concerns throughout the illness trajectory Adjusting treatment according to patients’ symptoms and concerns	Optimized information flow facilitates tailoring of remote caring practices
Barriers to technology use Challenges with interpersonal relationships due to virtual communication Difficulties summarizing complex symptoms and concerns	Technology, relationships, and complexity as perpetual obstacles in telehealth

### Potential for a Support System and Self-governance at Home

An advantage of telehealth is that the technology endorses patients’ access to care and improves their contact with HCPs, thus providing them with a support system at home [42,46,66,69,72,74,76-78,80], leading to feelings of security, safety, and closeness and connectedness with HCPs [46,52,66,76]. Just knowing that HCPs were available and looking out for them was emphasized by patients as important, reassuring, and meaningful [48,52,67,72,74,76]. Patients elaborated that the technology became a reminder of the support that is available to them [48,52].

The use of technology facilitated access to PC services at home, enabling patients to remain in their homes, being cared for and dying at home, rather than being hospitalized or traveling to outpatient clinics [42,43,47,50,52,65,66,70,75]. Being in the familiar home environment with access to remotely provided PC facilitates self-governance, as patients felt alive, relaxed, comfortable, and in control of their own lives [42,48,50,52,75]. The provided PC services ameliorate burdens, such as taxing symptoms and exhaustion, fear of infections after chemotherapy, practical arrangements, travel expenses, and long waiting times in hospitals, that are associated with travel-to-hospital consultations [47,48,50,52,69,71,75,80]. In contrast, some patients expressed that they did not feel that the use of telehealth prevented unnecessary hospital admissions [60]. One study indicated no significant differences in the number of hospital admissions between patients receiving teleconsultations with HCPs and the control group [53].

Patients expressed that substantial advantages of the technology were that it was both easy to learn how to use and to use [45,46,58,60,61,66,67,69,71,72,74,77,81,82] and that the technology was user-friendly, useful, and beneficial for their care [46,47,54,63,64,66,67,74,82]. Patients described that using the technology at home promoted feelings of satisfaction and comfort [47,48,53,57,58,60,63,68,69,71,72,74,75,79]. They did not experience the technology as onerous, burdensome, or time consuming [44,58,69,72,81,82], and they would recommend telehealth use to others [60,63,67,69,74,75,81,82]. Nonetheless, some patients also reported that telehealth was a challenge because communication became more intense and intrusive than telephone consultations [45,76].

Patients had different views regarding when it would be advantageous to introduce telehealth during their illness trajectory [45,49,77]. Some underlined that it would have been a greater advantage if telehealth had been introduced earlier or extended longer into their illness trajectory [77], whereas others expressed that technology would be an advantage only at certain stages of their illness or treatment [45,49].

### Visibility Supports Interpersonal Relationships and a Joint Understanding of Care Needs

One of the advantages of telehealth was that, over time, the remote interaction using videoconferences’ visual cues enabled patients to acquaint with the HCPs and build supportive and trusting interpersonal relationships with them [42,47,48,50,75,76]. Patients perceived that such relationships improved HCPs’ understanding of their care needs [42,48,76,78] and that a close connection with HCPs promoted patients’ feelings of being valued as unique persons and, to some extent, alleviated a sense of loneliness [47]. Patients experienced that HCPs provided emotional support and showed empathy via videoconferences by assessing their unique situations and visibly (verbally and nonverbally) responded to emotions, choosing considered care interventions over pity [42,80].

Patients expressed that videoconferences facilitated clearer communication and improved support, which was advantageous for both patients and HCPs [42,45,47,48]. Seeing each other’s facial expressions and eyes enhanced the ability to interpret visual cues and better understand each other. This made the patients who were receiving therapy through technology feel like a person within a context, rather than a patient in a hospital [48]. Other patients described that they felt safe when they saw the HCPs and that this enhanced the possibility to ask questions to clarify misunderstandings [45]. Others elaborated that, through the use of video, it was possible to observe the collaboration between HCPs, when 2 or more professionals were present, which was important because it strengthened the trust and confidence in care planning [47]. However, a few patients reported limited opportunities to see body language via videoconferences [79], whereas others stated that it was difficult to discuss certain topics when they did not see each other’s faces and suggested that including videos could enhance interaction and interpersonal dynamics [61].

Patients quickly lost the conscious awareness of technology during videoconferences with HCPs, and the conversations felt natural and meaningful. Telehealth became a time slot in which patients could discuss their problems rather than thinking about them at all hours [48]. Videoconferences also created a sense of distance and acted as a protective shield between patients and HCPs, facilitating the freedom to talk and helping patients express emotions and disclose personal information in a safe space [42,48,50,79]. Patients also expressed that videoconferences enhanced their confidence in asking questions during interactions [50].

Patients experienced that the use of videoconferences enhanced HCPs’ abilities to detect, assess, and understand their care needs [45,47,70,76-78] and that it was an advantage that HCPs could see them and their circumstances at home [42,48], which was not possible when talking with HCPs on the phone. During videoconferences, patients showed HCPs their physical changes, such as the development of nodes, and burdensome symptoms, such as struggling with breathlessness [47,76]. Furthermore, the use of video allowed HCPs to observe patients’ lives at home, such as mobility or bedsores, and to address questions and doubts regarding prognosis, treatment, and emotional support [70].

### Optimized Information Flow Facilitates Tailoring of Remote Caring Practices

Another advantage of telehealth was that patient participation using technology to self-report symptoms provided HCPs with information about their current symptoms and concerns [47,52,54,57,64,66,67,70,72,74]. This provided patients with a feeling of not being alone [52]. The technology functioned as a screening tool, facilitated reflection on patients’ perceptions of symptoms and whether medications or other self-management strategies were needed, and lowered the threshold for reporting symptoms [46,47,52]. Patients often integrated self-reporting into their daily routine [66], and the technology enabled them to self-report concerns that are beyond physical and psychological symptoms, including existential and spiritual concerns, emotions, and well-being [54,55,57,67,70,72]. Positive symptoms and joyful information were also reported, for example, good mood, being blessed, comfortable, at peace, happy, or performing meaningful activities [54,55,72]. Patients highlighted that the questions regarding the spiritual dimensions were appropriate [67].

Patients self-reported routinely on their symptoms, functional level, and concerns [47,64,66], although they were reluctant to do so at times [47]. There was no agreement among patients regarding the appropriate frequency of self-reporting [64,67,74]. Some patients expressed that longitudinal data may be more meaningful for HCPs than data from a single time point [44]. During the first 2 weeks of using telehealth, patients adhered more to the expectations of daily self-reporting of symptoms, which then decreased. Patients stopped self-reporting on average around 2 weeks before death [64].

Patients experienced that close monitoring of their symptoms and concerns was an advantage that enabled HCPs to detect and manage illness deterioration and potential problems and prioritize their caseloads [44,56,58,63,66,74]. The illness deterioration in some patients was detected at an early stage via telehealth, which led to treatment at home rather than hospitalization [66]. However, technology did not always detect illness deterioration, as symptoms developed between consultations [56].

Patients expressed that HCPs used information from their self-reporting to tailor and implement interventions during teleconsultation, telephone consultation, and home follow-up to discuss symptoms and treatment, adjust medications, provide advice, and offer outpatient clinic visits or hospitalizations [56,58,66,70]. Patients expressed that they had the possibility to participate in treatment decision-making and were pleased with the added possibilities, given the telehealth options [52]. However, it was a challenge for some patients in that the number of interventions declined when the illness deteriorated, and a patient who had triggered high-priority notifications even died before HCPs came to review the interventions [56]. In addition, in 1 study, there was no significant difference in the mean number of reported unmet needs between the patients who had received teleconsultations with HCPs and those who had received usual care [53].

### Technology, Relationships, and Complexity as Perpetual Obstacles in Telehealth

Patients experienced usability challenges [46-48,54,57,58, 62,66-68,70,74,77,80-82]. Insufficient knowledge, insufficient understanding of or unfamiliarity with the technology, as well as lack of clear instructions from HCPs, were obstacles to technology use [46,51,52,57,68,70,74,81]. Patients reported that they found telehealth overwhelming [51], struggled to learn to use the new technology, were concerned about making mistakes or destroying the device, and were worried about not being in control of the technology [81]. Clear instructions from HCPs on the use of telehealth at the initial appointment were essential for patients to continue using the technology [46]. Instead of using telehealth, some patients contacted HCPs directly [67], whereas others did not perceive a need to use technology, as their need for support was met outside the telehealth service [48]. In some studies, older age was a barrier to technology use because older patients doubted the usability of the technology, used it less often, and reported significantly lower scores regarding computer use acceptability than younger patients [46,62,67,74]. In contrast, some older patients had no difficulties using the technology [46,58]. Older patients expressed that younger patients would find the use of telehealth easier, but over time, these older patients felt a sense of mastery [50].

Patients experienced technical difficulties regarding the screen size, unclear images, and audio quality and feedback [44,45,61,70,80]. Difficulties with the internet, such as low bandwidth, led to communication problems [42,52,63,70,74,80,81]. Furthermore, patients disliked seeing their own image on the screen during videoconferences, which created feelings of self-consciousness and made the conversations feel less natural; however, for some patients, this challenge faded over time [48,80]. Patients also experienced equipment or software failure of the remote medical measuring devices [60,66,67,74,77]. Some patients were not able to use the remote medical measuring devices owing to technical problems [77]. Some patients were too ill to use technology and withdrew from the study or became unable to use technology owing to severe illness deterioration over time [54,66,74,81]. Other patients were too weak, tired, or unwell or had physical limitations such as tremors or reduced visual capacity to use the technology by themselves and needed help from their family members to manage measurements and report symptoms and concerns [47,66,67,81]. However, in a study, the majority of patients with motor neuron disease were able to use the application independently [81]. The use of a large computer for teleconsultation for bedbound patients as well as the use of an iPad for self-reporting of symptoms and functions were experienced by a few patients as antagonizing and burdensome, reminding them of their illness and impending death [42,47,52,82]. In contrast, other patients felt that self-reporting using telehealth was a positive challenge that allowed shifting the focus from pain to technology [74].

A challenge expressed by patients was that telehealth could not entirely replace the depth and quality of in-person care because of the nature and importance of human relationships, interactions, conversations, and the therapeutic value of in-person appointments with HCPs [47,49,71,72]. Patients also pointed out the importance of in-person care for establishing a relationship with HCPs [79] or for examining physically to detect changes such as weight loss, depression, and apathy, which patients could hide while communicating through electronic messages [49]. In 1 study, patients reported that they preferred a service that combined face-to-face and telehealth consultations [68], whereas in other studies, patients stated that video consultations were superior or equivalent to in-person or telephone conversations [75,79]. However, patients who had regular telephone contact with HCPs were significantly more satisfied with HCPs’ responses to questions and concerns than those who had used a mobile app and were only contacted by HCPs when their symptoms escalated [55].

Another communication challenge threatening interpersonal relationships was communicating via videoconferencing with several HCPs simultaneously [43,45,47,49]. Patients expressed that it was challenging to complete videoconferences without interruptions when several HCPs participated and that it would be easier to know when to talk or be quiet during in-person meetings [45]. Others became insecure when HCPs disagreed openly, and patients took the role of mediator between HCPs during the videoconferences [43]. The use of telehealth could violate the patients’ privacy [42,66,69], for example, if other HCPs entered the hospital videoconference room unannounced during videoconferences, interrupting conversations. In contrast, other patients did not feel that telehealth interfered with their privacy [58,75].

Patients experienced communication challenges with summarizing complex and fluctuating symptoms and concerns when they self-reported using numerical ratings [44,47,54,64,66,67,74], whereas some patients preferred numeric ratings rather than yes-or-no responses [67]. Although the use of word clouds with qualitative descriptions of pain and a body map for pain location were perceived as helpful to some extent, it was challenging for patients to summarize various types of coexisting pain [44]. Furthermore, patients wanted to self-report the quality, nature, and emotional impact of their symptoms and concerns and wanted HCPs to know the full extent of what they were going through [44,47,74]. To elaborate on their numeric ratings, patients took an active role and responsibility by sending text messages or using the free text option on the technology to provide HCPs with additional information [44,47,54,64]. Such information included pain locations, burdensome symptoms that were not included in the technology, factors triggering the pain, and detailed descriptions of how changes in medications affected their symptoms [44,54,64].

Patients had different experiences of whether the use of telehealth contributed to symptom relief and optimal quality of life. Some patients reported substantial challenges of increased burdensome symptoms and reduced quality of life from using telehealth [52,58-60,70,72], whereas this was not the case for other patients [53-55,58,73,75]. Moreover, some patients reported increased burdensome symptoms, such as fear and anxiety or higher symptom severity, as a challenge after using telehealth [53,61].

The technology for self-reporting had several limitations, such as a lack of open-ended questions for the description of pain, adverse effects, and triggers for breakthrough pain; no possibility of reporting pain in different body locations; and lack of a section to summarize the significant events of past week and a graphic view that could help patients compare how symptoms had developed over days, weeks, and months [44,47,74]. Patients saw no value in self-reporting when they had no symptoms or felt that their condition would not improve after self-reporting [52]. Some became bored because of daily self-reporting on the same questions, whereas others thought that variation would make things more complicated [74]. Not receiving any response on their self-reporting made the patients question whether anyone had read it [52].

## Discussion

### Principal Findings

This systematic mixed studies review aimed to critically appraise and synthesize the findings from studies that investigated patients’ use of telehealth in home-based PC. The advantages of telehealth included the technology’s potential as a support system that enabled patients to remain at home and how the visual features of telehealth enabled patients to build interpersonal relationships with HCPs over time. In addition, self-reporting provided HCPs with information about patients’ symptoms and circumstances, allowing for more tailored care for specific patients. Challenges with the use of telehealth were related to barriers in usability, technical issues, and inflexible reporting of complex and fluctuating symptoms and circumstances in the electronic questionnaires for self-reporting. Telehealth was also described as intrusive and a threat to privacy at home.

Being able to stay at home, rather than visiting the outpatient clinic or being hospitalized, was a major advantage for many patients using telehealth. Subsequently, patients’ self-governance in their daily lives was promoted and maintained. A systematic review suggested that telehealth may increase patient autonomy [83]. From a person-centered PC approach, autonomy and self-determination focus on patients’ need to be active participants in their care and allow them to govern their lives according to their values, preferences, and beliefs [4]. Having choice and control could promote patients’ feelings of normalcy, maintaining their own identities while living with a life-threatening illness [84]. Patients prefer to be cared for in the care setting of their choice, which is important for their quality of life, along with PC services that align with their preferences, providing care continuity, help, or support when needed [6,84].

However, 2 of the studies [53,60] included in our systematic mixed studies review contradicted that the use of telehealth prevents hospitalization. This has previously been described in a systematic review regarding the use of telehealth in PC in the United Kingdom [85]. Although there seems to be avoidable reasons for transferring patients from the home to a hospital, some hospitalizations are necessary as patients with PC needs may be in need of physical examinations and interventions that cannot be performed at home [86] or administered through telehealth. In some cases, the patients may prefer to be cared for in an institution rather than at home [6].

PC has been characterized as “high-touch rather than high-tech” [20], and concerns have been raised regarding the tension between the potential for technology to improve care and the extent to which technology may affect the relationship between patients with PC needs and HCPs [21]. In line with a previous systematic review [28], our findings indicate that an advantage of using telehealth is that videoconferences facilitate nuances in communication, which patients in turn found helpful in building supportive and trusting interpersonal relationships with HCPs. This seems to facilitate a joint understanding of patients’ circumstances. Furthermore, the findings of our systematic mixed studies review also emphasize the importance of remote *interaction over time* between patients and HCPs to build such relationships. A continuous relationship seems to be paramount and may not be linked to a particular HCP, but rather to a unified approach in the HCP team responsible for follow-up [87]. From a person-centered PC approach, relationships in which HCPs are attentive, listening, and validating experiences are important for patients to share their stories and express their needs [4]. The use of videoconferencing could allow patients and HCPs to interact in a nuanced and personal manner, including subtle visual cues [28]. However, in some of the studies included in our review [47,49,71,72], patients experienced that telehealth could not replace the depth and quality of personal interactions. This finding suggests that a combined service of telehealth and in-person care would be preferable for many patients. For example, having an initial face-to-face meeting, regardless of care modality for follow-up, as the HCPs in a PC team suggest [88], could be one flexible approach that is well aligned with person-centered PC [4].

Symptom management is a prerequisite in PC for preserving patients’ self-image (identity), well-being, and quality of life, as well as facilitating their ability to continue with the daily activities they enjoy [4,84]. Our findings suggest that patient participation using telehealth to self-report symptoms and concerns optimized the information exchange with HCPs. This facilitated HCPs’ ability to detect and manage deteriorations and problems and to tailor care interventions. Embracing a person-centered PC approach [4] is paramount, and our findings suggest that the information sharing enabled patients to participate in the cocreation of their care owing to their ability and possibility to report what they perceived as the most bothersome symptoms and concerns. In addition, because PC is being integrated earlier in the illness trajectory, when patients are more able to self-report [89], it is important to provide appropriate infrastructure to ensure the actual use of telehealth technology. Poor-quality electronic questionnaires, for example, that have an overload or overlap of questions or require reporting of symptoms that patients find irrelevant, may affect patients’ acceptability and use of remote self-reporting [90]. Our findings showed that several patients highlighted that space for free text and elaboration was important for self-reporting. A continuous dialogue between patients with PC needs and HCPs is a prerequisite to identifying symptoms and concerns that are meaningful and relevant for patients to self-report during the PC trajectory, from early PC to end-of-life care. Receiving feedback or a response when reporting symptoms would be helpful for patients to “feel” heard, otherwise they may feel like they are communicating, but no one is listening.

PC addresses issues related to pain and other problems, whether physical, psychosocial, existential, or spiritual [1]. In many of the studies in our review, patients self-reported physiological and psychological symptoms, whereas only a few studies included patients self-reporting existential or spiritual concerns, emotions, and well-being. This may be owing to the challenges in quantifying such concerns [21]; however, patients’ unmet needs in the psychological domain, and specifically, emotional support was highlighted [91]. Hence, including patients and families in care planning is imperative. In line with the findings of our systematic mixed studies review, future research should address how data from mobile devices can be used for the remote monitoring of and caring for emotional well-being [32].

Our findings suggest that patients experienced several technology-related obstacles, such as insufficient technology literacy and difficulty in learning and using new technology. Although technical literacy, usability concerns, and lack of technical support seem to be frequent barriers to telehealth use among older adults [83,92], some of the older patients in our systematic mixed studies review experienced no challenges with technology. Fostering and building patients’ telehealth capabilities may be important for the success and viability of remote home care, particularly for older patients and patients who are less familiar with technology [24].

We identified other challenges, such as difficulties related to slow internet connection, reduced audio and image quality, telehealth design, difficulties in telehealth use owing to illness deterioration or physical limitations, and difficulties in self-reporting complex symptoms using numeric ratings. However, some of the included studies possibly used technology for remote monitoring that may be outdated, whereas more recent studies used smartphones or tablet technology, which can be easier and more intuitive to use. Telehealth is growing [85] and with the development and implementation of new technologies, some of these challenges may be mitigated. For instance, to facilitate remote communication between older patients with cancer and their families and friends, designers have developed a larger tablet with only one button, tailored for use by older people with limited technological literacy [93].

The challenges identified in our review should be addressed in the further development of existing and new technologies. Telehealth technology needs to be relevant for patients in the PC setting, as the user needs and requirements may differ from those of the general population. The needs and requirements of the intended population should drive the design and development, usability, and functions of telehealth technology [32]. The technology needs to suit patients, not vice versa [94]. Co-design may be a key approach to delivering person-centered care, as it allows for the involvement of the intended users, such as patients and HCPs, in the development of telehealth technology [95]. This could be a way to align technology to patients’ needs and requirements, subsequently increasing the sustainability and usefulness, given patients’ mixed experiences with regard to depth and quality of relationships as well as experience of intrusion and threatened privacy. The inclusion of intended users from the beginning of the design process, sufficient assessment of the needs and capabilities of intended users, and usability testing and early feedback from intended users through prototype testing are essential to help telehealth designers and developers address telehealth patient experience throughout the entire design process [94].

### Strengths and Limitations

The strength of our systematic mixed studies review lies in its methodological rigor. The review was conducted according to an acknowledged methodological framework; the systematic search was conducted in several databases; and the search strategy was developed in close collaboration with an experienced research librarian and peer-reviewed by a second librarian. The study selection, quality assessment, and data extraction were conducted systematically and in parallel by 2 independent researchers. The first author analyzed the data by developing codes and themes. The second and the last author independently read the codes and the descriptive and analytical themes, asked critical questions, and provided competing interpretations. This was an iterative process and revisions were made accordingly. The final analytical themes were determined through consensus proceedings among all the authors. This facilitated reflexivity, dependability, credibility, and intersubjectivity.

A limitation could be that we may not have been able to identify all potential search terms for telehealth and PC because there are several synonyms used for these terms. Our systematic mixed studies review also had some language restrictions. There may be studies published in other languages that we were not able to identify. Another limitation could be that we did not address the differences or similarities between the advantages and challenges of different telehealth modalities when we synthesized the data. Some of our findings may relate more to one modality than another. The transferability of our findings may be limited by the fact that most of the studies were conducted in resource-rich countries and many of the studies comprised patients with cancer diagnoses. The results should also be interpreted with caution because of clinical and methodological heterogeneity among the included studies.

### Conclusions

Telehealth provides patients with a remotely managed support system at home, which could enable them to stay at home rather than be hospitalized. The visual features enhance remote communication, enabling patients to build interpersonal and trusting relationships with HCPs over time. Patients’ self-reporting of symptoms and concerns optimizes the information flow and exchanges with HCPs. Challenges with the use of telehealth are related to technological barriers, such as technology illiteracy, technical issues, telehealth technology design, and difficulties using telehealth technology owing to illness deterioration or physical limitations. Only a few studies have included telehealth services that enabled patients to self-report existential or spiritual concerns, emotions, and well-being. Telehealth was perceived as intrusive and a threat to privacy at home by some patients.

Our findings can be used to guide the further development of existing services and development of new telehealth technology in home-based PC. The challenges that patients experience with telehealth use underline the importance of including patients with PC needs as intended users in the design process. The inclusion of intended users seems paramount to developing a telehealth service that patients perceive as both meaningful and relevant, which may be of particular importance when patients face limited life expectancy.

Future research should investigate patients’ experiences of self-reporting of existential and spiritual concerns, emotions, and well-being, as well as the effectiveness of telehealth to meet such concerns.

## Appendix

Multimedia Appendix 1 PRISMA 2020 Checklist.

Multimedia Appendix 2 Deviations from the published protocol.

Multimedia Appendix 3 Search strategy used in Medline.

Multimedia Appendix 4 Characteristic of the included studies.

Multimedia Appendix 5 Assessment of methodological quality of the included studies using Mixed Methods Appraisal Tool.

## Abbreviations

HCP: health care professional
PC: palliative care
PRISMA: Preferred Reporting Items for Systematic Reviews and Meta-Analyses
