# Supplementary material for: Advantages and Challenges of Using Telehealth for Home-Based Palliative Care: Systematic Mixed Studies Review
Source: J Med Internet Res. 2023 Mar 13;25:e43684. doi: 10.2196/43684 (PMC10131904; doi:10.2196/43684)
Supplement: Multimedia Appendix 2 [file jmir_v25i1e43684_app2.docx]

| **Approach described in the protocol** | **Description and justification of the**  **deviation from protocol** |
| --- | --- |
| The results will be discussed in light of the technology acceptance model owing to its importance of understanding what influences patients’ acceptance of technology. Our review could contribute recommendations for practice and policy, enabling the implementation of patient-centered telehealth services that align with patient preferences, needs, and values. | After synthesizing the data, we deemed that it would be more relevant and suitable to discuss the results in light of theory regarding person centered palliative care. |
| Joanna Brigs Institute appraisal tools to assess methodological quality. | To reduce the number of checklist due to several studied design we deemed that it would be more beneficial to use the Mixed methods appraisal tools which is an appraisal tool designed for the appraisal stage of systematic mixed studies review. |
| Data were extracted from the included reports using a standardized data collection form that included the following data: Year of publication; country of origin; aim of the study; study population and sample size; theoretical framework for the telehealth intervention; telehealth application; design and methods; and findings related to the research questions of the review. | After peer review, data regarding telehealth delivery mode was also extracted |
| Data from the results section of the included papers will be extracted independently by pairs of reviewers. | The first author uploaded the PDFs for the included papers in NVivo, and then result section of reach included report was line by line coded using NVvio. |

**Multimedia Appendix 2.** Deviations from the published protocol.
