# Supplementary material for: Advantages and Challenges of Using Telehealth for Home-Based Palliative Care: Systematic Mixed Studies Review
Source: J Med Internet Res. 2023 Mar 13;25:e43684. doi: 10.2196/43684 (PMC10131904; doi:10.2196/43684)
Supplement: Multimedia Appendix 3 [file jmir_v25i1e43684_app3.docx]

**Multimedia Appendix 3.** Search strategy used in Medline.

| Search number | Mesh term, text word and combinations |
| --- | --- |
| 1 | exp Palliative Care/ (53844) |
| 2 | exp Palliative Medicine/ (345) |
| 3 | exp "Hospice and Palliative Care Nursing"/ (866) |
| 4 | exp Terminal Care/ (51092) |
| 5 | exp Hospice Care/ (6454) |
| 6 | exp Terminally Ill/ (6496) |
| 7 | (palliat* or dying* or hospice* or "end of life" or "supportive car*" or "life limiting illness*").kf,tw. (140447) |
| 8 | (terminal* adj2 (ill* or care* or caring* or treatment* or period* or nurs* or patient*)).kf,tw. (13418) |
| 9 | exp Telemedicine/ (28334) |
| 10 | exp Videoconferencing/ (1820) |
| 11 | exp Telerehabilitation/ (361) |
| 12 | exp After-Hours Care/ (1866) |
| 13 | exp Mobile Applications/ (5811) |
| 14 | exp Remote Consultation/ (4787) |
| 15 | exp Webcasts as Topic/ (333) |
| 16 | exp Telecommunications/ (91424) |
| 17 | exp Wireless Technology/ (3543) |
| 18 | exp Cell Phone/ (10608) |
| 19 | exp Computers/ (77918) |
| 20 | exp Computers, Handheld/ (7807) |
| 21 | (((Web or video) adj2 (seminar* or conferenc* or consult* or cast* or application*1)) or ((wearable or wireless) adj2 (technolog* or electronic*)) or ((digital* or technolog*) adj3 health) or ((remote* or internet or electronic* or robot*) adj2 (care* or cari* or consultati* or application*1)) or (assist* adj2 (living or technolog*))).kf,tw. (49932) |
| 22 | (telemedicin* or "tele medicin*" or telehealth* or "tele health*" or telecare* or "tele care*" or telecari* or "tele cari*" or telecommunicat* or "tele communicat*" or teleconferenc* or "tele conferenc*" or teleconsultat* or "tele consultat*" or telenurs* or "tele nurs*" or telemonitor* or "tele monitor*" or teletherap* or "tele therap*" or telerehab* or "tele rehab*" or App*1 or "app-base*" or mobile* or phon* or telephon* or Android* or tablet* or iphon* or Skyp* or Zoom* or ehealth* or "e health*" or emedic* or "e medic*" or erehab* or "e rehab*" or mhealth* or "m health*" or ehomecare* or "e homecare*" or "e home care*" or ehomecari* or "e homecari*" or "e home cari*" or "smart home*" or smarthome* or smartphon* or cellphon* or "personal digital assistant" or pda or "palm pilot*" or "after hours car*" or "out of hours car*" or "Short Message Service" or SMS or textmessag* or "text messag*" or texting or "distance support" or videoconferenc* or webconferenc* or webcast* or computer* or webinar*).kf,tw. (643903) |
| 23 | 9 or 10 or 11 or 12 or 13 or 14 or 15 or 16 or 17 or 18 or 19 or 20 or 21 or 22 (772005) |
| 24 | 1 or 2 or 3 or 4 or 5 or 6 or 7 or 8 (187784) |
| 25 | 23 and 24 (4555) |
| 26 | limit 25 to yr="2010-Current" (2589) |
