# Supplementary material for: Advantages and Challenges of Using Telehealth for Home-Based Palliative Care: Systematic Mixed Studies Review
Source: J Med Internet Res. 2023 Mar 13;25:e43684. doi: 10.2196/43684 (PMC10131904; doi:10.2196/43684)
Supplement: Multimedia Appendix 4 [file jmir_v25i1e43684_app4.docx]

**Multimedia Appendix 4.** Characteristic of the included studies.

| **Author, year, country** | **Aim** | **Study population and sample size** | **Theoretical framework** | **Telehealth application, delivered mode** | **Design and methods** | **Findings** |
| --- | --- | --- | --- | --- | --- | --- |
| Adam et al. [44] (2020)  UK | To develop a novel theory and evidence-based digital intervention to optimise cancer pain control in the community | 2 patients (cancer); 1 male, age range 55-73 years | Intervention mapping approach of six steps allowing behaviour change theory to be applied systematically to a health problem | A digital app (Can-Pain) used by patients who are prescribed strong opioids, and a consultation with a HCP^1^ in which data generated by the app can give allow HCPs to give patients tailored feedback about pain management.  *Remote monitoring, asynchronous mode* | Feasibility testing  Individual telephone interviews | Patients felt that being closely monitored was an advantage and that their monitoring reports would help HCPs to effectively prioritise and recognise problems. Patients felt that longitudinal symptom data might be more meaningful to HCPs than assessments at a single point |
| Ando et al. [56] (2019)  UK | To develop questions for Careportal and then to evaluate the use of the questions as a function of telemonitoring in the care of MND^2^ on non-invasive ventilation | 13 patients (MND) anticipated survival >= 6 moths; 8 males, mean age 66 (range 49-73) years. 10 patients completed the pilot study | NR^3^ | Tablet based remote monitoring device (Careportal) of symptoms for timely care. Patients answered questions and transferred nocturnal pulse oximetry readings once a week via Careportal. Monitoring by a non-clinical researcher and physiotherapist.  *Remote monitoring, asynchronous mode* | 24-week pilot study evaluation and intervention | 61 interventions were made (10 patients) including: arranging a new appointment (n=12); treatment adjustment (n=16); equipment provision (n=19); referral (n=14). Blood oxygen saturation levels were maintained, time ventilated and inspiratory pressures increased during the trial |
| Bandini et al. [78] (2022)  US | To examine and compare in-person versus telehealth experience among outpatient palliative care programs and patients | 187 patients (diagnosis NR) used only video, sex NR, age NR | NR | Video follow-up.  *Video, synchronous mode* | Mixed-mode survey | Patients expressed that it was easy use video to get care from palliative care HCPs. Patients felt heard and understood, that HCPs put their best interest first, saw them as a person and understood what was important to them |
| Bernat et al. [57] (2015)  US | To pilot an abbreviated DT^4^ intervention using a legacy-building web portal for adults with terminal cancer | 16 patients (terminal cancer); 4 males, mean age 52 (SD^4^ =12) years | Dignity Model  DT | A face-to-face DT session that included audio recording each participant's responses to three (out of nine) DT questions. Respondents used a novel web portal to respond to the remaining six DT questions with text and/or other materials (e.g., photographs). Participants received brief weekly facilitative calls from the interventionist to add flexibility to the more rigid structure of a web-based intervention.  *Remote written, asynchronous mode* | Pilot feasibility and acceptability study  Baseline and post-intervention surveys assessing primary outcome (feasibility and acceptability) and secondary (existential wellbeing and dignity)  Descriptive statistics | Participant satisfaction was high with the intervention and the final legacy project they created. Less than half of the participants reported using the web portal to complete their legacy project; the majority used a word processing program. Of those who used the portal, 80% reported dissatisfaction, primarily due to usability issues and a lack of clear instructions |
| Besse et al. [58] (2016)  The Netherland | To evaluate the feasibility of SMS^6^ and IVR^7^to improve pain management in patients with cancer, including terminally ill patients | 17 patients (cancer); 7 males mean age 58 (range 27-75) years | NR | Mobile phone. The use of SMS and IVR. During four weeks the patients received daily IVR to provide pains scores on a NRS^8^ using their mobile phone, and based on the score a nurse would contact the patient if action was needed.  *Remote monitoring, asynchronous mode* | Feasibility study  QoL^9^: the EORTC QLQ-C30^10^ Questionnaire before and after the intervention  Satisfaction: questionnaire (0-10)  Descriptive statistics | All patients were satisfied with the intervention. No difficulties for the, mainly older, patients in handling this communication way on pain intensity. Pain scores declined during the study |
| Bonsignore et al. [72] (2018)  US | To evaluate the feasibility and acceptability of a telehealth program in a rural palliative care population | 101 patients (22 pulmonary, 19 cancer, 14 neurologic, 14 cardiovascular, 5 renal, 3 gastrointestinal, 12 other, 11 unknown); 40 males, mean age 72 years | NR | TapCloud remote patient monitoring application and videoconferencing designed to connect patients and providers outside the clinical setting. Patients have access to individualized care plan, reminders, symptom review and medication management.  HCPs have access to a clinical dashboard monitored twice daily.  *Remote monitoring, asynchronous mode, and video, synchronous mode* | A mixed-methods evaluation approach to assess feasibility, usability, acceptability  Quantitative using a quality data collection assessment tool  Qualitative: Semi-structured interviews | The top 2 symptoms taped were positive symptoms: good mood and comfortable. Top 3 negative symptoms included back pain, tired, short of breath. Patients perceived that TapCloud enhanced access to HCPs, made medication refill easy and gave a deep sense of comfort, knowing that HCPs was a tap away. Tap-Cloud was not able to replace the depth of in-person care |
| Calton et al. [79] (2020)  US | To characterize the experience of patients with serious illness and their caregivers receiving palliative care by telemedicine | 35 patients (17 cancer, 8 pulmonary, 6 liver, 4 neurologic,1 immunologic); 20 males, mean age 61 (range 27-83) years | NR | Videoconferencing with HCPs from palliative care outpatient practise.  *Video, synchronous mode* | Mixed methods Telephone survey  Descriptive statistics  Thematic analysis | Patients reported satisfaction and that felt telemedicine was an acceptable format to discuss most sensitive topics. Patients had concerns about rapport building and desired a more user-friendly telemedicine platform |
| Funderskov et al. [45] (2019)  Denmark | To explore the use of video consultations, experienced by patients and their relatives, as part of SPC^11^ at home | 11 patients (cancer); 7 males, mean age 59 (range 30-68) years | NR | Telephone contact with the SPC team nurse, approximately once a week video consultation was set by the SPC nurse and the patients. A tablet was used for one-way calls between patients, relatives, and the SPC team nurse. An app allowed relatives to participate in group consultations.  *Video, synchronous mode* | Explorative qualitative study  Participant observations, semi-structured interviews  Giorgi’s descriptive phenomenological method | Patients found video consultations feasible but did not agree on when to using it in relation to their physical condition. Patients with limited ability to speak experienced great support using the visual communication. Numbers of participants could affect the video consultation if too many join |
| Eastman et al. [68] (2021)  Australia | To understand patient and HCPs perspectives on the use of telehealth within community palliative care prac- tice | 74 patients (diagnosis NR); 42 males, median age 75 years | NR | Interaction lasting > 10 minutes between patient and palliative care HCP using a telecommunication device (video or telephone).  *Video, synchronous mode* | Survey | Patients felt comfortable using telehealth, but experienced technological issues and felt under educated using the system. Patients preferred community palliative care service that combined face-to-face and telehealth |
| Geronimo et al. [69] (2017)  US | To assess the feasibility and acceptability of telehealth for ALS^12^ care via real-time videoconferencing from the clinic to patients’ homes | 11 patients (ALS); 7 males, median 60 (range 34-80) years | NR | Polycom RealPrescence system for videoconferencing between patient’s device at home and nurse and physician using a dedicated ALS clinic computer. HCPs participated individually or in peers to interact with patients.  *Video, synchronous mode* | Survey | Videoconferencing allowed for good communication, description of care, provision of care recommendations, and removed burden of travel. Patients reported lack of privacy, technological issues and the feeling of missing out on clinic |
| Hackett et al. [46] (2020)  UK | To report HCP and patients’ perceptions of their engagement with an ICT^13^ system for pain management to understand the mechanisms that could support implementation of ICT-based interventions into routine palliative care practice | 12 patients (cancer) recruited from the intervention arm of a RCT^14^; age NR, sex NR | NR | Access to PainCheck (ICT system) for routine pain assessment and monitoring and a self-management educational booklet and DVD^14^ (Tackling Cancer Pain).  *Remote monitoring, asynchronous mode* | Qualitative study  Semi-structured interviews  Thematic analysis | PainCheck was perceived as simple to complete and beneficial to care. PainCheck resulted in feeling more connected to a support system, enabling them to reflect on their pain and its management and reminded patients to engage in self-management strategies |
| Hebert et al. [73] (2016)  Canada | To compare conventional palliative homecare visits and a combination of conventional and home telehealth ‘video-visits’ with respect to symptom management and QoL^15^ | IG^16^: 25 patients (diagnosis NR); 9 males mean age 70 (range 43-89) years  CG^17^: 19 patients (diagnosis NR); 7 males, mean age 63 (range 36-94) years | NR | IG: Video visits in combination with conventional palliative care homecare visits.  *Video, synchronous mode*  CG: Conventional palliative care homecare visits | Multi-method study  RCT  Semi-structured interviews  Thematic analysis | No significant differences between the groups in measures of symptom measures and QoL. Patients generally indicated a higher level of readiness to use the technology than home care nurses |
| Helleman et al. [82] (2020)  The Netherlands | To evaluate the use of telehealth as part of specialized care for patients with ALS and the user experiences of patients and HCP | 50 patients (ALS); 32 males, mean age 61.4 (SD 13.0) years | NR | ALS Home-monitoring and coaching: Application-based self-monitoring on tablet, smartphone or PC; message function, alerts, monthly follow-up by nurse practitioner via e- or telephone consultation.  *Remote monitoring, asynchronous mode* | Multi-method prospective single center cohort study  Data application  Online surveys  Semi-structured interviews  Descriptive statistics  Thematic analysis | Most patients experienced that telehealth was easy, helpful, not burdensome, and reported satisfaction with flexible consultations |
| Hennemann-Krause et al. [70] (2015)  Brazil | To examine telemedicine as a form of home and additional support for  traditional outpatient care as a way to remotely monitor and manage the symptoms of patients  with advanced cancer | 12 patients (cancer); 7 men, mean age 68 (SD=9.43) years | NR | In-person consultations at the hospital with multidisciplinary team (physician, nurse, social worker, psychologist, and music therapist) monthly. *Web* conferencing weekly with multidisciplinary team, electronic messaging (email), telephone calls, ICTs, and home visits.  *Remote monitoring, synchronous mode and video, synchronous mode* | Prospective, longitudinal, qualitative, descriptive, and case series study  ESAS^18^ used to assess symptoms  Descriptive statistics | Of the 8 patients who completed the ESAS in both in-person and remote contact 6 had lower symptom scores in the web conference interviews than in person |
| Hermosilla-Ávila et al. [59] (2019)  Chile | To assess the effect of an intervention of humanized nursing accompaniment, at home, on the QoL of people with advanced cancer and of their family caregivers | 17 patients (cancer), sex NR, age NR | Watson’s theory of human caring and subjective living experience | Nursing counseling included 3 home visits per month, *on-line counseling* with educational platform supported by audio visual contents through an internet platform on web, tablet or mobile to provide general information and specific aspects requested by patients, telephone contact for 3 months.  *Video, synchronous mode* | Pre-experimental study with pre- and post-intervention measurements | The post-test showed improvement in global health and decrease of symptoms such as fatigue, dyspnea, constipation and anorexia |
| Hobson et al. [81] (2019)  UK | To evaluate the processes involved in using a novel digitally enabled healthcare system TiM^19^ in people living with MND and their informal carers | IG: 20 patients (MND); 14 males, mean age 60.4 (SD 11.7, range 30-78) years  CG: 20 patients (MND); 14 males, mean age 60.0 (SD 10.0, range 39-73) years | NR | IG: Patient app on tablet containing patient questions assessing disease progression, complications of MND, use of medical interventions and patient well-being that were completed weekly or more frequently. Results were sent to MND nurse that reviewed the results and provide telephone advice.  *Remote monitoring, asynchronous mode*  CG: Usual care | Multi-method pilot and feasibility RCT  Patient reported  outcome measures using questionnaires  Semi-structured interviews  Thematic analysis  Triangulation compare quantitative and qualitative data | App was acceptable and accessible for patients |
| Hochstenbach et al. [74] (2016)  The Netherlands | To evaluate the feasibility of a mobile and web-based intervention to support self-management in outpatients with cancer pain | 11 patients (cancer) with moderate to severe pain; 5 males, mean age 53 (SD=15) years | Self-management | The intervention involved daily monitoring, graphical feedback, education, and advice using a mobile application on iPad 2 for patients and a web application for nurses.  *Remote monitoring, asynchronous mode* | Multi-method feasibility study  20-item questionnaire to assess learnability, usability, desirability  Descriptive statistics  Semis-structured interviews | Patients quickly learned how to manage the app, easily used the different components, and liked to work with the app. The app was considered simple, clear and required limited energy. Patients experienced comfort using the app |
| Hoek et al. [53] (2017)  The Netherlands | To determine whether weekly teleconsultations from a hospital-based SPCT^20^ improved patient-experienced symptom burden compared to “care as usual” and to  determine the effects of these teleconsultations on unmet palliative care needs, continuity of care and hospital admissions | IG: 38 patients (cancer) with life-expectancy of ≤ 3 months; 71% males, mean age 62 (SD= 9.0) years  CG: 36 patients (cancer) with life-expectancy of ≤ 3 months; 61% males, mean age 62 (SD=10.6) years | NR | IG: Weekly prescheduled teleconsultations for 12-weeks period with an SPCT-member (physician or nurse) using a desktop/tablet in addition to usual care. GP^20^ were invited to join the patient during the teleconsultation.  *Video, synchronous mode*  CG: Usual care included palliative home care provided by GP, supported by SPCT according to the standard referral procedures, by phone or by patients visiting the outpatient clinic | Two armed, non-blinded RCT | The IG reported higher symptom burden than the CG. The number of unmet needs, experienced continuity of care, and reported hospital admissions did not differ between the groups |
| Hutchinson et al. [80] (2022)  US | To explore the acceptability, feasibility, and emotional responsiveness of telemedicine delivered palliative care | 11 patients (7 heart failure, 2 pulmonary disease, 1, neurologic disease, 1 end-stage liver disease); 5 males, mean age 70.8 (SD 13.3) years | NR | Telemedicine delivered palliative care video consultations by nurse practitioner and palliative care physicians.  *Video, synchronous mode* | Mixed-methods pilot study  Questionnaires  Semi-structures interviews  Video coding | All patients rated video visits as equal to or better to than in-person visits to provide emotional support. Video consultations improved access to care, reduced preparation for visits and the added values. Disadvantages were technical issues, and impact of seeing oneself |
| Jiang et al. [75] (2020)  Australia | To assess the feasibility of integrating telehealth-assisted home-based specialist palliative care into a rural community setting | IG: 14 patients (10 cancer, non-cancer); 7 males,  mean age 66 (SD=15.6) years  CG: 7 patients (5 cancer, 2 non-cancer); 2 males, mean age 79 (SD=8.8) years | NR | IG: Initial video consultations (Web Real Time Communication Platform) HCP and caregiver. Video consultation to patient with nurse visiting patient home. PC physicians provided medical correspondence to other HCPs involved. Patients encouraged to request follow-up video consultation. Standard care.  *Video, synchronous mode*  CG: Standard care: Support from general medicine physicians, district nurses, palliative-care nurse practitioners and local GP. Patients could only access HCP indirectly by a monthly multidisciplinary meeting and by telephone with a metropolitan-located palliative-care physician | Prospective mixed methods pilot  Primary outcomes:  time efficiency of the service and user satisfaction  Palliative Care Outcomes Collaboration measures | Patient feedback showed a high level of overall satisfaction. The IG showed less functional decline, and functional status remained better than the CG. The IG had fewer community palliative care nurse visits, fewer GP visits and fewer hospital admissions than the CG |
| Keenan et al. [50] (2021)  UK | To explore potential divergence and convergence in relation to health care professionals’ and patients’ acceptability of the use of telehealth within palliative care provision through the lens of Self-Determination Theory | 3 patients (1 breast cancer, 1 lung cancer, 1bone cancer); 0 males, mean age NR (range 48-72) years | Self-Determination Theory | Psychosocial support from therapist part of the palliative care team via *video* on laptop computers.  *Video, synchronous mode* | Qualitative approach  Semi-structured interviews  Deductive thematic analysis | Patients expressed that telehealth could create a more autonomy supportive environment, reduced travel burden, provided freedom to plan their day, increased confidence in asking question in interaction with HCP. At baseline patients expressed that younger patients would find accessing support using telehealth, but over time these patients felt mastery |
| Lind, Carlgren & Karlsson [66] (2016)  Sweden | To explore  whether the health diary system and method could improve  patient self-management, support symptom control, decrease  acute hospital readmissions, and thereby contribute to improve the quality of care | 14 patients (severe heart failure); 11 males, median age 84 (range 75-95) years | NR | Digital pain and health diary forms; green (symptoms, weight, medication) and blue (measurements) forms, possible to send free text messages to HCP. Responsible physicians assess reported data. All patients informed that they telephone clinic if necessary.  *Remote monitoring, asynchronous mode* | Pilot study | The technology was accepted by patients who experienced an improved contact with HCP, felt more compliant with HCPs’ advice, and felt more secure and more involved in their own care |
| Lind & Karlson [54] (2013)  Sweden | To investigate if the use of the PallPen system could speed up the symptom assessment retrieval in order to facilitate and support symptom control in palliative end-of-life home care. | IG: 13 patients (cancer); 8 males, mean age 64 (range 51-78) years  CG: 9 patients (cancer); 5 males, mean age 69 (range 42-87) years | NR | IG: Digital pen technology (PallPen), and ICT system for receiving, storing, and presenting information to nurses. Digital symptom diary on digital paper and usual care.  *Remote monitoring, asynchronous mode*  CG: Usual care including care from specialized home care centres via home visit or telephone. Used non digital pen and symptom diary on printed paper | Two armed RCT | There was a significant shorter median time span from reporting to noticing for assessment in the IG than CG. No significant differences between the groups regarding median assessed pain, median wellbeing or median number of rescue doses of analgesic |
| Middleton-Green et al. [76] (2019)  UK | To evaluate “Gold Line”, a 24/7, nurse led telephone and video-consultation support service for patients thought to be in the last year of life | 8 patients; 2 patients had 2 or more life-limiting conditions, 4 males, aged 43-88 years | Gold Standards Framework | Gold Line is a 24/7 point of contact for patients provided by senior nurses via telephone, or a video app on an iPad. Referral to the service enables the team to access patients’ full electronic health record.  *Video, synchronous mode* | Multi-method  Quantitative data from patient records  Semi-structured interviews  Analysed for board themes | Patients reported the emotional support and advice they received at difficult times and the value of knowing that Gold Line existing in knowing it was there. Some felt the iPad offered additional benefits in terms of face- to face contact |
| Morgan et al. [47] (2017)  Australia | To discuss and demonstrate how patients and carers can engage in telehealth supported palliative care | 1 patient (cancer), male, 57 years old | NR | Self-reporting of function and symptoms via applications on iPad and video conferencing with nursing staff/GP via iPad. Predefined thresholds triggered email alert to palliative care community nurse.  *Remote monitoring,* a*synchronous mode, and video, synchronous mode* | Case report | Self-reporting was perceived as a means to communicate symptoms without bother to the community palliative care team. Video-conferencing with the nurse and GP was valued as an effective way to communicate and eliminated the need for travel |
| Nemecek et al. [60] (2019)  Austria | To investigate the effect of telemedical care on QoL in patients with advanced cancer and their family caregivers and if telemedicine decreases the number of hospital admissions | IG: 8 patients (cancer); sex NR, age NR  CG: 7 patients (cancer); sex NR, age NR | NR | IG: iPad Minis and “VSee” video application enabled patients and caregivers to send direct message to responsible physician and ask for medical advice around the clock, communicate with physician using videoconferencing, register patient`s vital signs and ongoing treatment  *Remote monitoring, synchronous mode, and video, synchronous mode*  CG: Usual care NR | A controlled feasibility study | Significant lower scores on the hospital anxiety and depression scale in the IG than CG, suggesting improved QoL. A decrease in the number of hospital admissions could not be shown. The user satisfaction was very good |
| Ngomoa et al. [55] (2021)  Tanzania | To assess the effectiveness of a smartphone- or Web-based app, mPCL^22^, to extend specialist access via shared data and communication with local health workers | IG: 49 patients (cancer); 9 males; mean age NR  CG: (49 patients (cancer); 18 males; mean age NR | NR | IG: Patients got in-person training on use of mPCL on *android device* or *desktop device* to complete and submit POS, mobile communication with specialist or local HCPs in response to worrisome POS^23^ and in-person assessment by local HCP or palliative care nurse in response to escalating symptoms.  *Remote monitoring, asynchronous mode*  CG: Phone-contact: Received calls twice a week from study personnel that collected POS | Pilot RCT | Symptom severity was significantly lower in the phone-contact group and decreased over time in both groups however, between-group change in overall symptoms over time did not vary significantly. Care satisfaction was generally high in both groups |
| Pasanen et al. [51] (2022)  Australia | To qualitatively explore the experiences of both palliative care physicians and patients settings, including their views on its future role in health care | 11 patients (11 cancer); 5 males, age 62 (range 36-74) years | NR | Medical consultation delivered via telephone or audio-/video-link  *Video, synchronous mode* | Exploratory qualitative design | Unless family or carers were present to facilitate the video-ink may patients preferred to telephone consultation |
| Phillip et al. [71] (2022)  Australia | To evaluate patient and physician satisfaction, acceptability, and utility of outpatient palliative care provision through telehealth | 127 patients (118 advanced cancer, 1 neurodegenerative, 6 renal failure, 1 other); 55 males, mean age 63.5 (range 52-70) years | NR | Telehealth appointment delivered through video and/or telephone  *Video, synchronous mode* | Multi-site prospective, cross-sectional observational study | 75% of the patients assessed the telehealth appointment as satisfactory. Compared with the use of audio alone, use of both video and audio media were associated with higher scores regarding greater usefulness, ease of use and learnability, satisfaction, and future use of telehealth |
| Pinto et al. [67] (2017)  Portugal | To assess the feasibility and acceptability of the Comfort app prototype | 7 patients (1 multiple sclerosis, 2 amyotrophic sclerosis, 4 cancer; 5 males, mean age 40 (range 27-54) years | NR | The comfort app can be used via smartphone, tablet or computer to assess 11 self-reported items including symptoms, fear of the future and will to live  *Remote monitoring, asynchronous mode* | Feasibility study | Patients considered app to be very simple, clear and easy to use. Patients preferred using tablet/computers due to larger font size on these devices. That someone was looking out for them and what they were feeling was important |
| Rahman et al. [48] (2020)  UK | To explore the experiences of rural palliative care patients receiving psychosocial support through telehealth | 3 patients (cancer); 0 males, aged 48-72 years | NR | Laptop at home to access psychological support via videoconferencing (Polycom RealPresence video conferencing system) from psychotherapist in general hospital weekly over 3 months.  *Video, synchronous mode* | Qualitative design with longitudinal interpretive phenomenological approach  Semi structured interviews  Longitudinal interpretive phenomenological analysis | Patients developed a relationship with the psychotherapist. The distance created by the technology facilitated a freedom to talk. Being aware of physical images on screen created feelings of self-consciousness and removed the naturalness of the conversation |
| Reinke et al. [61] (2011)  US | To explore whether educating patients via Web conferencing  (webinar) would equip them with knowledge and skills to engage in conversations about EoL^24^ care | 7 patients (COPD^25^); 4 males, mean age 68 (SD= 4, range 64-72) years | NR | A 60-minute interactive webinar on end-of-life communication; Adobe Connect Pro Live used as webinar platform; research assistant provided technology training for participants prior to webinar; attendee listen and chat function.  *Audio and written, synchronous mode* | Single group pre-post test study  Pre-survey, post-survey, 3 months post seminar assessment of changes in EoL care planning  Descriptive statistics | Technology was easy to use. Patients felt the webinar was an acceptable option if unable to participate in person. All voiced that adding a video stream would have promoted interaction in the context of these sensitive conversations. At 3 months, all participants had taken further action on EoL planning |
| Schoppee et al. [62] (2019)  US | To examine computer use acceptability  scores of patients with end-stage cancer in hospice and their caregivers and to compare  the scores for differences by age, gender, race, and computer use experience | 234 patients (cancer) experienced pain ≥ 3 in past 24 hrs; 49% males, mean age 69 (SD=14.0, range 20-100) years | Computer-human interface design evaluation; focus on ease of use, intuitive, aesthetically pleasing, and understandable | Patients receiving home hospice care reported pain, once daily, for one week using PAINReportIt via a Wi-Fi-enabled tablet.  *Remote monitoring,* a*synchronous mode* | Pre-post test study  CAS^26^ questionnaire comprising. Descriptive and inferential statistics | Patient CAS score ranged 5-14 (max 14; mean 12.2, SD 1.9). Older patients had statistically significant lower cores than younger patients. Patients who reported never using computers had the lowest CAS scores |
| Slavin-Stewart et al. [63] (2020)  Canada | To evaluate the use of the FaceTime application on an iPad to improve timely access to physician consultation for home-based palliative care patients living  in rural Nova Scotia | 15 patients (cancer); 7 males, mean age 69 (55-88) years, palliative performance mean score 53 (30-70) | NR | Use of FaceTime on iPad Air 2 for videoconference meetings. Visits were triggered by change in clinical status. Patient, lay caregiver, nurse and physician in video-conference meetings.  *Video, synchronous mode* | Observational, exploratory study  Paper survey to assess patient satisfaction with consultation  Descriptive statistics | The audio and visual quality allowed patients to communicate easily with the doctor. No consultations were terminated due to network instability. Patients found the FaceTime encounter highly acceptable. All stated that their medical concerns were addressed and were willing to use FaceTime again |
| Stern et al. [77] (2012)  Canada | To explore the perceptions of family caregivers and palliative cancer patients of  home telehealth, and their experience with it | 11 patients (cancer); 7 males, median age 61 (range 34–91) years | NR | Nurses with expertise in palliative care communicated with patients and lay caregivers via telephone or *videophone*; *remote monitoring* (blood pressure, blood oxygen levels and heart, lung, abdominal sounds). Outcomes triggered physical visit.  *Remote monitoring, asynchronous mode, and video, synchronous mode* | Mixed-methods case study  Quantitative data for nursing charts, Qualitative interviews, direct observations Descriptive statistics Thematic content analysis | Families stressed how important easy access to HCPs was. Most common reason for contact with tele-nurse was pain (55%). Technical issues with remote monitoring device. Patients perceived that equipment was easy to use but needed assistance with blood pressure cuff application |
| Teter et al. [65] (2021)  US | To examine the impact of accessibility  to a provider via telemedicine on emergency depart-ment visit rates in adults, 35 years and older, on home hospice and palliative care | IG: 22 patients (10 cardiopulmonary, 5 cancer, 4 Alzheimer, 1 diabetes, 1 liver disease, 1 neurological); 7 males, mean age 70.5 (SD 11.9) years  CG: 22 patients (8 cardiopulmonary, 4 cancer, 5 Alzheimer, 2 diabetes, 3 neurological); 11 males, mean 73.8 (SD 12.7) years | NR | IG: Web-based application that allowed audio communication and video visits. Patients could contact HCP using application when in need of the HCP regardless if symptomatic, was ongoing.  *Video, synchronous mode* | Quasi-experimental | There were significantly fewer emergency departments visits and 911 calls in the IG |
| Tieman et al. [64] (2016)  Australia | To assess the feasibility of a telehealth-based model of service  provision for community-based palliative care patients, carers and clinicians | 43 patients (41 cancer); 61% males, mean age 72 (range 49-91) years | NR | Telehealth model for specialist palliative homecare using iPad: (1) videoconferencing clinicians, patients, caregivers, (2) case conferences between patient, clinician, GP, (3) electronic self-report assessment tools, (4) remote monitoring. Alerts to HCP if self-reported data alarming; expected to enter data both daily and weekly.  *Remote monitoring, asynchronous mode, and video synchronous mode* | Prospective cohort study Australian-modified Karnofsky Performance Scale, Assessment of QoL, and Symptom Assessment Scale  Descriptive statistics | All patients managed to enter data  using the telehealth system. Self-reported data entered by patients identified changes in performance  status leading to changes in care. Over 4000 alerts were generated |
| Van Gurp et al. [42] (2015)  The Netherlands | To focus on the impact  of teleconsultation technologies on the relationships between home-based palliative care patients, GP SPCT | 18 patients (16 cancer, 2 COPD) with life expectancy ≤ 3 months and karnofsky score ≤ 60; 10 males, mean age 61 (range 24-86) years | NR | Weekly teleconsultations between hospital based SPCT, palliative care patients and GP invited to join in patients’ homes. 2 teleconsultation devices were uses: desktop computer (“Bidibox”) and iPad 2.  *Video, synchronous mode* | Qualitative, observational design Serial semi-structured interviews  Grounded theory approach | Technology enabled specialist care at home; visual features facilitated connectedness but could jeopardize patient integrity. Aspects of technology influenced quality care; like if broadband was weak, interactions suffered. Technology fitted into domestic lives of patients; teleconsultation enabled long-term engagement; availability of specialists promoted sense of safety and relief |
| Van Gurp et al. [43] (2016)  The Netherlands | To describe whether and how teleconsultation supports the integration of primary care, specialist palliative  care, and patient perspectives and services and how patients and (in)formal caregivers experience collaboration in a teleconsultation  approach | 18 patients (16 cancer, 2 COPD) with life expectancy ≤ 3 months and karnofsky score ≤ 60; 10 males, mean age 61 (range 24-86) years | NR | Weekly *teleconsultations* between hospital based SPCT, palliative care patients and GP invited to join in patients’ homes. 2 teleconsultation devices were uses: desktop computer (“Bidibox”) and iPad 2.  *Video, synchronous mode* | Qualitative, observational design Serial semi-structured interviews  Grounded theory approach | Some patients complained  about the insecurity that resulted from witnessing professionals moving in contradictory directions, with the patient serving as a mediator between both |
| Viitala et al. [52] (2021)  Finland | To examine patients’ experiences regarding the impact of support given by a mobile application on their ability to cope with incurable cancer | 20 patients (12 breast cancer, 7 gastrointestinal, 1 Lymphoma); 5 males, median years 54 (range 31-74) years | NR | Smart cloud-based mobile application for patient-reported outcomes regarding symptoms.  Remote monitoring, *asynchronous mode* | Qualitative approach  Semi-structured interviews  Inductive content analysis | Most patients experienced application as helpful to monitor relevant symptoms and coping with their disease and that application increased their ability to contact HCP. A few patients reported a disease-centredness in using the application |
| Voruganti et al. [49] (2018)  Canada | To understand participant perceptions on electronic communication in general  and the added value of the new tool in particular | 6 patients (cancer); 3 males, mean age 60 (range 42-85) years | Constructivist approach which asserts that reality is a social construction, influenced by social and historical context | A three-month pilot intervention evaluating electronic, web-based communication tool called Loop; tool connects HCP, patients and lay caregivers in secure virtual environment. Purpose to facilitate interprofessional collaboration in setting goals and reaching goals.  *Remote written, asynchronous mode* | Qualitative, descriptive Individual interviews 1-4 times  Thematic analysis using NVivo | Electronic communication was good for certain things. Concerns about email over traditional approaches reflected awareness of provider business. With Loop, patients received more timely answer to questions between appointments, the asynchronous nature of the tool reduced «telephone tag», gave time to more carefully formulated questions, and allowed the amalgamation of information outside of appointment time |

^1^HCP: Healthcare Professional; ^2^MND: motor neuron disease ^3^NR: Not Reported; ^4^DT: Dignity Therapy; ^5^SD: Standard Deviation; ^6^SMS: Short Message Service; ^7^IVR: Interactive Voice Response; ^8^NRS: Numeric Rating Scale; ^9^QoL: Quality of Life ^10^EORTC QLQ-C30: European Organization for Research and Treatment of Cancer Quality of Life Questionnaire; ^11^SPC: Specialized Palliative Care; ^12^ALS: amyotrophic lateral sclerosis; ^13^ICT: Information and Communications Technology; ^14^RCT: Randomized Controlled Trial; ^15^DVD: Digital Video Disc; ^16^IG: Intervention Group: ^17^CG: Control Group; ^18^ ESAS: Edmonton Symptom Assessment System; ^19^TiM: telehealth in motor neuron disease; ^20^GP: General Practitioners; ^21^SPCT: Specialist Palliative Care Consultation Team; ^22^mPCL; mPalliative Care Link; ^23^POS: Palliative care Outcome Scale; ^24^EoL: end-of-life; ^25^COPD: Chronic Obstructive Pulmonary Disease; ^20^CAS: Computer Acceptability Scale;
