# Supplementary material for: Advantages and Challenges of Using Telehealth for Home-Based Palliative Care: Systematic Mixed Studies Review
Source: J Med Internet Res. 2023 Mar 13;25:e43684. doi: 10.2196/43684 (PMC10131904; doi:10.2196/43684)
Supplement: Multimedia Appendix 5 [file jmir_v25i1e43684_app5.docx]

**Multimedia Appendix 5.** Assessment of methodological quality of the included studies using Mixed Methods Appraisal Tool.

| **Assessment** | **Authors** | **Clear research questions?** | **Do the collected data allow to address the research questions?** | **Qualitative approach appropriate to answer the research question?** | **Are the qualitative data collection methods adequate to address the research question?** | **Findings adequately derived from the data?** | **Interpretation of results sufficiently substantiated by data?** | **Coherence between qualitative data sources, collection, analysis and interpretation?** | |
| --- | --- | --- | --- | --- | --- | --- | --- | --- | --- |
| Qualitative studies | Adam et al. [44] | Yes (Y) | Y | Y | Y | Y | Y | Y | |
|  | Funderskov et al. [45] | Y | Y | Y | Y | Y | Y | Y | |
|  | Hackett et al. [46] | Y | Y | Y | Can’t tell (C) | C | Y | Y | |
|  | Keenan et al. [50] | Y | Y | Y | Y | Y | Y | Y | |
|  | Morgan et al. [47] | C | C | Y | No (N) | N | C | N | |
|  | Pasanen et al. [51] | Y | Y | Y | Y | Y | Y | Y | |
|  | Rahman et al. [48] | Y | Y | Y | Y | Y | Y | Y | |
|  | van Gurp et al. [42] | Y | Y | Y | Y | Y | Y | Y | |
|  | van Gurp et al. [43] | Y | Y | Y | Y | Y | Y | Y | |
|  | Vitala et al. [52] | Y | Y | Y | Y | Y | Y | Y | |
|  | Voruganti et al. [49] | Y | Y | Y | Y | Y | Y | Y | |
| **Assessment** | **Authors** | **Clear research questions?** | **Do the collected data allow to address the research questions?** | **Randomization appropriately performed?** | **Groups comparable at baseline?** | **Complete outcome data** | **Outcome assessors blinded to the intervention provided?** | **Participants adhere to the assigned intervention?** | |
| Quantitative randomized controlled trials | Hoek et al. [53] | Y | Y | Y | Y | N | N | N | |
|  | Lind et al. [54] | Y | Y | C | C | Y | C | C | |
|  | Ngoma et al. [55] | Y | Y | Y | N | Y | N | C | |
| **Assessment** | **Author, year** | **Clear research questions?** | **Do the collected data allow to address the research questions?** | **Participants representative of the target population?** | **Measurements appropriate regarding both the outcome and intervention (or exposure)?** | **Complete outcome data?** | **Confounders accounted for in the design and analysis?** | **During the study period, is the intervention administered (or exposure occurred) as intended?** | |
| Quantitative non-randomized studies | Ando et al. [56] | Y | Y | Y | Y | Y | Y | Y | |
|  | Bernat et al.  [57] | Y | Y | Y | Y | Y | N | N | |
|  | Besse et al. [58] | Y | Y | Y | Y | Y | C | Y | |
|  | Hermosilla-Ávila et al. [59] | Y | Y | Y | Y | Y | N | Y | |
|  | Nemeck et al. [60] | Y | Y | C | C | Y | C | C | |
|  | Reinke et al. [61] | Y | Y | Y | Y | C | C | C | |
|  | Schoppee et al. [62] | Y | Y | Y | Y | Y | C | Y | |
|  | Slavin-Stewart et al. [63] | Y | Y | Y | Y | Y | C | Y | |
|  | Tiemann et al. [64] | Y | Y | Y | Y | N | C | Y | |
|  | Teter et al. [65] | Y | Y | Y | Y | Y | C | Y | |
|  | Lind et al. [66] | Y | Y | Y | Y | Y | C | Y | |
|  | Pinto et al. [67] | Y | Y | Y | Y | Y | C | Y | |
| **Assessment** | **Authors** | **Clear research questions?** | **Do the collected data allow to address the research questions?** | **Sampling strategy relevant to address the research question?** | **Sample representative of the target population?** | **Measurements appropriate?** | **The risk of nonresponse bias low?** | **Statistical analysis appropriate to answer the research question?** | |
| Quantitative descriptive studies | Eastman et al. [68] | Y | Y | Y | Y | Y | N | Y | |
|  | Geronimo et al. [69] | Y | Y | Y | Y | Y | N | Y | |
|  | Hennemann-Krause et al. [70] | Y | Y | Y | Y | Y | Y | Y | |
|  | Philip et al. [71] | Y | Y | Y | Y | Y | Y | Y | |
| Assessment | **Authors** | **Clear research questions?** | **Do the collected data allow to address the research questions?** | **Adequate rationale for using a mixed methods design to address the research question?** | **The different components of the study effectively integrated to answer the research question?** | **Outputs of the integration of qualitative and quantitative components adequately interpreted?** | **Divergences and inconsistencies between quantitative and qualitative results adequately addressed?** | **The different components of the study adhere to the quality criteria of each tradition of the methods involved?** |  |
| Mixed methods studies | Bandini et al. [78] | Y | Y | Y | N | N | N | Y | |
|  | Bonsignore et al. [72] | Y | Y | Y | N | Y | Y | Y | |
|  | Calton et al. [79] | Y | Y | N | Y | Y | N | Y | |
|  | Hebert et al. [73] | Y | Y | N | Y | N | N | N | |
|  | Hobson et al. [81] | Y | Y | Y | Y | Y | Y | Y | |
|  | Helleman et al. [82] | Y | Y | Y | N | N | N | Y | |
|  | Hochstenbach et al. [74] | Y | Y | Y | Y | Y | Y | Y | |
|  | Hutchinson et al. [80] | Y | Y | Y | N | Y | Y | N | |
|  | Jiang et al. [75] | Y | Y | C | C | N | N | N | |
|  | Middleton-Green et al. [76] | Y | Y | C | C | N | N | N | |
|  | Stern et al. [77] | Y | Y | C | N | N | N | Y | |
